# MicroRNAs expressed in depression and their associated pathways: A systematic review and a bioinformatics analysis

**DOI:** 10.1016/j.jchemneu.2019.101650

**Published:** 2019-10

**Authors:** Camila Perelló Ferrúa, Roberta Giorgi, Laísa Camerini da Rosa, Cainá Corrêa do Amaral, Gabriele Cordenonzi Ghisleni, Ricardo Tavares Pinheiro, Fernanda Nedel

**Keywords:** microRNAs, Depressive disorder, Systematic review, Bioinformatic, Signaling pathway

## Abstract

Depression is a debilitating mental illness, one of the most prevalent worldwide. MicroRNAs have been studied to better understand the biological mechanisms that regulate this disease. This study review systematically the literature to identify which microRNAs are currently being associated with depression and their related pathways. The electronic search was conducted in PubMed, Scopus, Scielo, ISI Web of Knowledge, and PsycINFO databases, using the search terms “Depressive Disorder” or “Depression” and “MicroRNAs”. After, microRNAs that were up and down-regulated in depression were analyzed by bioinformatics. We observed that among the 77 microRNAs cited by included studies, 54 had their levels altered in depressed individuals compared to controls, 30 being up-regulated and 24 down-regulated. The bioinformatics analysis revealed that among the up-regulated microRNAs there were 81 total and 43 union pathways, with 15 presenting a significant difference. Among the down-regulated microRNAs, 67 total and 45 union pathways were found, with 14 presenting a significant difference. The miR-17-5p and let-7a-5p were the most frequently found microRNAs in the statistically significant pathways. In this study a panel of altered microRNAs in depression was created with their related pathways, which is a step towards understanding the complex network of microRNAs in depression.

## Highlights

• MicroRNAs are altered in depressed patients.
• 54 microRNAs were altered in blood sample from depressive patients.
• Bioinformatics analysis revealed 29 pathways related with microRNAs in depression.
• miR-17-5p and let-7a-5p were the most frequent in the statistically significant pathways.

## 1 Introduction

Depression is a chronic and debilitating mental illness (Smith, 2014) characterized by sadness, absence of interest and pleasure, sleep and appetite disorders, poor concentration, tiredness, guilt, and low self-esteem (APA, 2000). Among mental disorders, depression is one of the most prevalent worldwide (Baxter et al., 2013). According to the World Health Organization, more than 300 million people suffer from depression and the disease is estimated to become the second cause of disability (after cancer) worldwide in the near future (WHO, 2017). Therefore, it is relevant to better understand the biological mechanisms that regulate this disease.

MicroRNAs are small molecules of non-coding RNA formed of about 23 nucleotides. They present unique sequences that binds to the 3′ untranslated region (UTR) of their targets mRNAs causing translational repression or destabilization, and consequently reducing the overall output of associated proteins (He and Hannon, 2004). Therefore, microRNAs play an important role in regulatory function, both in normal physiological activities (Dwivedi, 2011) – including cell cycle, apoptosis and neuronal development (Vreugdenhil and Berezikov, 2010), and intracellular pathway signaling (Rivera-Barahona et al., 2017) – and in the development of disease (Zhang et al., 2017; Sebastiani et al., 2017; Wan et al., 2015) – including cancer (Zhang et al., 2017; Esquela-Kerscher and Slack, 2006), diabetes (Sebastiani et al., 2017), and neuropsychiatric disorders, such as depression (Sebastiani et al., 2017). However, this regulatory capacity of microRNAs is not confined to the cell or tissue of origin. MicroRNAs can travel in biological fluids - serum (Wan et al., 2015), plasma (Camkurt et al., 2015), urine (Mari-Alexandre et al., 2016; Maluf et al., 2014), cerebrospinal fluid and saliva (Xie et al., 2013; Lipschitz et al., 2013) - exerting their activity in distal cells or tissues, promoting endocrine gene regulation (Valadi et al., 2007). The circulating microRNAs, therefore, have to ensure their stability in order to travel through the biological fluids. This is afforded in great part by the association of microRNAs in exosomes (Zernecke et al., 2009) and high-density lipoproteins (Vickers et al., 2011), protecting and preventing degradation before reaching the target cells.

Circulating microRNAs have been constantly evaluated as indicators of pathologic condition, and their use to identify depression or depressive symptoms is not an exception. In this sense, evidence suggests that there is a relationship between circulating microRNAs and the development of depression (Dwivedi, 2014). Alterations of microRNAs involved in pathways associated with affective disorders seem to trigger and aggravate this disease (Mouillet-Richard et al., 2012). Recently, a case-control study reported that patients with depression have lower levels of brain-derived neurotrophic factor (BDNF) and increased expression of microRNA-132 and microRNA-182, when compared with healthy subjects, suggesting that serum BDNF and the related microRNAs can be used as biomarkers for diagnosis or as therapeutic targets of depression (Li et al., 2013). In another study with depressed subjects, plasma microRNA-320a was significantly down-regulated and microRNA-451a was significantly up-regulated when compared to healthy controls (Camkurt et al., 2015).

It is currently known that many peripheral blood microRNAs undergo expression changes when confronted with the challenge of depression, including microRNA-338, microRNA-1244 (Fan et al., 2014), microRNA-335, microRNA-583 (Li et al., 2015), microRNA-508-3p (Smalheiser et al., 2014), microRNA-16 (Song et al., 2015; Baudry et al., 2010), microRNA-34a-5p, microRNA-221-3p (Wan et al., 2015), among others. However, the absence of information, especially of top quality scientific evidence, reveals that mental disorders such as depression are still of low priority compared to other global health problems (Baxter et al., 2013).

Thus, the aim of this study was to systematically review the literature to identify which microRNAs are currently being associated with depression and their related pathways.

## 2 Material and methods

### 2.1 Review questions

Which microRNAs have been related to depression?

In which pathways do these microRNAs act?

### 2.2 Inclusion and exclusion criteria

The inclusion criterion for article assessment was being a case control study on depression in humans assessing microRNA expression. The exclusion criteria were literature reviews, studies not conducted in humans, congress summaries, patent descriptions, book sections, hypothesis articles, commentaries, studies that are not case and control, studies unrelated to depression, studies unrelated to microRNAs, studies in which the case group was treated with medication, methodological approaches, opinion articles, previews, expertise opinions, letters, news, and studies with confounding factors. Articles that were not fully available, even after attempting to contact the authors, were also excluded.

### 2.3 Search strategy

The electronic search was conducted without initial date restriction up to and including April 2017 in PubMed, Scopus, Scielo, ISI Web of Knowledge, and PsycINFO databases. Google Scholar database was not used in this case since microRNAs have been recently established, especially in relation to depression, and this database has been shown to have low precision (Boeker et al., 2013). Therefore its information could compromise our results.

An initial search was conducted using the following: ((((((("Depression"[Mesh]) OR "Depression"[all]) OR "Depressive Disorder"[Mesh]) OR "Depressive Disorder"[all])))) AND ((("MicroRNAs"[Mesh]) OR "MicroRNAs"[all])); TITLE-ABS-KEY ("Depression" OR "Depressive Disorder") AND TITLE-ABS-KEY("MicroRNAs"); (("Depression" OR "Depressive Disorder") AND ("MicroRNAs")); (("Depression" OR "Depressive Disorder") AND ("MicroRNAs")); (("Depression" OR "Depressive Disorder") AND ("MicroRNAs")). No language restriction was applied.

**Fig. 1:**
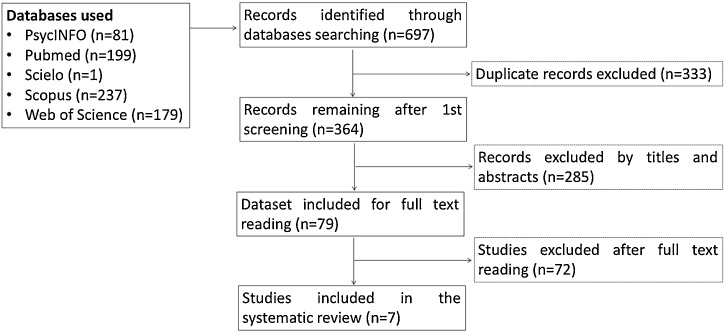
Flow diagram of study selection.

All references were managed in the EndNote X7 software (Thomson Reuters, New York, NY, US). Initially, duplicate references were excluded. Titles, abstracts, and study methodologies were screened based on the inclusion and exclusion criteria by two reviewers independently (CPF and LCdaR). Lists were compared and in case of disagreement, a consensus was reached by discussion. When a consensus was not achieved, a third reviewer decided if the article should be included (FN). This systematic review followed the PRISMA statements, with some adjustments (Fig. 1) (Moher et al., 2009).

### 2.4 Data extraction

Data were extracted and tabulated independently by two reviewers (CPF and LCdaR) to be submitted to a descriptive analysis. Cases of disagreement were handled as described above. A meta-analysis of the data was not feasible, given the absence of agreement in the literature to date.

### 2.5 Bioinformatics analysis

After extracting the data, microRNAs that were up and down-regulated in depression patients compared to controls were inserted separately, using the software DIANA Tools, mirPath v.3. The microRNAs that were not recognized by the software were disregarded in the bioinformatics analysis. Thus, the exclusion criterion for bioinformatics analyses was absence of investigated microRNAs indexed in mirPath v.3.

The microRNA functional analyses were performed in real-time using Kyoto Encyclopedia of Genes and Genoma (KEGG). The human species was selected to investigate the microRNAs. In the optional filter menu of expressed genes, no interaction was selected, and statistics were used for a subset of expressed genes. The interactions dataset chosen for all microRNAs was TarBase v7.0, based on results from previous positive and negative experiments. In the advanced statistics options, False Discovery Rate (FDR) correction was chosen. The Fisher’s Exact Test was used with a p-value threshold of 0.05. After inserting all microRNAs into the software, pathway unions (defined as the pathways containing more than one associated microRNA) were observed and significant ones were selected in the up-regulated and down-regulated microRNAs.

### 2.6 Quality assessment

The Newcastle - Ottawa Scale (NOS) was used by two independent researchers (CPF and RGSM) to assess the quality of the included case control studies. The scale uses a star system to assess three domains: selection, comparability, and exposure. Cases of disagreement were handled as described above.

A descriptive analysis was performed and studies that did not adequately describe the microRNAs and did not contain indexed microRNAs (in DIANA Tools) were considered of poor quality.

## 3 Results

The initial search yielded 697 articles; after removing 333 duplicate titles, a total of 364 articles were included for title and abstract screening after which 79 articles remained. After reading the full text, 71 studies were excluded. Of the 8 articles that satisfied the inclusion criteria, 7 reported data feasible for bioinformatics analysis. Fig. 1 displays the PRISMA flowchart for the study selection process.

Table 1 reports the clinical characteristics of depressive and control populations. Four studies were conducted in Asia (Wan et al., 2015; Camkurt et al., 2015; Li et al., 2015; Sun et al., 2016), one in North America (Leidinger et al., 2013), and two did not describe the ethnicity of the target population (Roy et al., 2017; Maffioletti et al., 2016). One study investigated depression only in female patients (Camkurt et al., 2015), five investigated both genders (Li et al., 2015; Sun et al., 2016; Leidinger et al., 2013; Maffioletti et al., 2016; Roy et al., 2017), and one did not mention the gender of the subjects under investigation (Wan et al., 2015).

**Table 1:** Main findings of the studies including sample size, age, gender, and nationality of depressive and control individuals.

Author and year of publication	N diseases	Target population diseases	Age diseases	Gender diseases	Nationality of depressive individuals	N control	Target population control	Age Control	Gender control	Nationality of control individuals
Camkurt et al., 2015 (16)	N = 50	People from Mersin University Teaching	36.94 ± 11.18	35 female	Turkish	N = 41	People from Mersin University Teaching	36.20 ± 8.54	27 female	Turkish
Leidinger et al., 2013 (26)	N total = 15 N RT-qPCR = 15	Patients from SAMPLE Registry of PrecisionMed (San Diego, CA, USA)	Mean ± SD 45.2 (9.1)	15 male	North americans	N Total = 22	Patients from ACE Registry, a PrecisionMed-UBC collaboration	Mean ± SD 67.1 (7.5)	11 male 11 female	North americans
Li et al., 2015 (18)	N = 18	Patients from the department of Psychiatry, the First Affiliated Hospital of Chongqin Hospital of Chongqing Medical University	N (%) 15.1 (2.8) Between 12 and 17	10 male 8 female	China	N = 18		N (%) 15.3 (3.1)	11 male 7 female	Chinese
Maffioletti et al., 2016(27)	N = 20		Mean ± SD 47.70 ± 11.91	3 male 17 female		N = 20		Mean ± SD 45.05 ± 10.79	5 male 15 female	
Roy et al., 2017 (28)	N = 18		45.44 ± 2.44	7 male 11 female		N = 17		38.47 ± 2.54	6 male 11 female	
Sun et al., 2016 (25)	N = 32	Patients First Hospital of Shanxi Medical University	Mean ± SD 40.5 ± 5.9	13 male (40.6%) 19 female (59.4%)	Han nationality	N = 32	Patients from First Hospital of Shanxi Medical University	Mean ± SD 38.3 ± 6.5	14 male (43.8%) 18 female (56.2%)	Han nationality Chinese
Wan et al., 2015 (11)	N total = 38 N Blood = 32		Mean ± SD Analysis group 32.0 ± 10.8 Validated group 34.33 ± 10.4	Analysis group 3 male 3 female Validated group 15 male 17 female		N total = 27 N Blood = 21		Mean ± SD Analysis group 32.5 ± 9.2 Validated group 35.34 ± 9.5	Analysis group 2 male 4 female Validated group 10 male 11 female	Singaporean

Table 2 shows the instruments used for the diagnosis of depression. Only one study did not mention this factor (Leidinger et al., 2013). The Diagnostic and Statistical Manual of Mental Disorders – IV (DSM-IV) was the most used among the studies. However, a great heterogeneity between the instruments used is evident.

**Table 2:** Main findings of the studies including instruments and scales for depression diagnosis, mean scores for depressive and control individuals and p values for the difference between groups (MINI: Mini International Neuropsychiatric Interview Plus; ICD-10: Diagnostic criteria for depression).

Author and year of publication	Instrument for depression diagnosis	Scale for depression diagnosis	Depressive scores	Control scores	p value
Camkurt et al., 2015 (16)	SCID with HDRS SCID with MADRS No comorbid psychiatric or medical conditions	Above 17 (SCID with HDRS) Above 20 (SCID with MADRS)	Median Std. Er. 19 Q3% (75 %)-Q1(25%) 17-22 Mean SD 28 5.05	Median Std. Er. 2 Q1(25%)-Q3(75%) 1-4 Mean SD 3.15 1.81	p < 0.001 p < 0.001
Leidinger et al., 2013 (26)					
Li et al., 2015 (18)	ICD-10 or DSM-IV HAM-D 17-item version		Score Mean: N(%) 25.27 (43.21)	Score Mean: N(%) 1.7 (1.1)	p<0.0001
Maffioletti et al., 2016(27)	DSM-IV-TR HAM-D17 SCID-I (to confirm diagnosis) MINI	Minimum total score of 14 on the 17-item (HAM-D17) Control: any medical diseases or in pharmacological treatment or has a BMI ≥ 30	Mean ± S.D. 23.60 ± 3.74		
Roy et al., 2017 (28)	MINI or SCID MADRS		34.05 ± 2.41	None	p = 0.06
Sun et al., 2016 (25)	DSM-IV and Chinese version of DSM-IV TR Axis I Disorders Patient Edition HAMD-17		Mean score ± SD 22.6 ± 3.4		

**Fig. 2:**
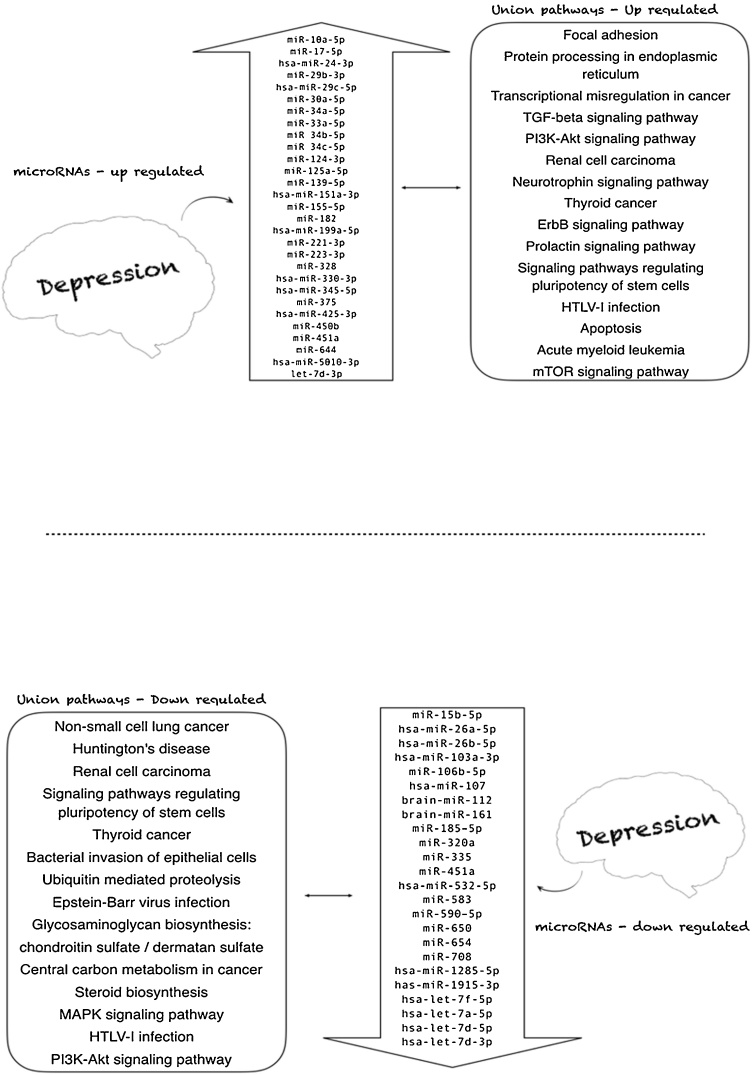
Panel of microRNAs up- and down-regulated, and pathways associated with depressed patients when compared to controls.

Table 3 and Fig. 2 report the main findings for microRNAs. We observed that among the 77 microRNAs cited by the seven included studies, 54 had their levels altered in depressed people compared to controls, 30 being up-regulated and 24 down-regulated (Fig. 2). All analyses were performed in blood, including plasma (Camkurt et al., 2015), leukocytes (Sun et al., 2016), and serum (Wan et al., 2015; Roy et al., 2017). However, some authors did not specify the blood component (Li et al., 2015; Leidinger et al., 2013; Maffioletti et al., 2016).

**Table 3:** Main findings of the studies for microRNA expression in depressed (N) and control subjects (N) analyzed by qrt-PCR, and the origin tissue. 1) up-regulated microRNAs, 2) down-regulated microRNAs and 3) unchanged microRNAs. The nomenclature of the tabulated microRNAs was transcribed exactly as mentioned in the included studies.

Author and year of publication	Size sample depressive	Size sample control	Up-regulated microRNAs^1^	Down-regulated microRNAs^2^	Unchanged microRNAs^3^	Origin tissue	Target microRNAs^b^
Camkurt et al., 2015 (16)	N = 50	N = 41	miR-451a miR-17-5p miR-223-3p	miR-320a	miR-25-3p miR-126-3p miR-16-5p miR-93-5p	Plasma	*GRIN2A* *DISC1* *SLC17A7*
Leidinger et al., 2013 (26)	N = 15	N = 21	hsa-miR-5010-3p hsa-miR-151a-3p	hsa-let-7f-5p hsa-miR-1285-5p hsa-miR-107 hsa-miR-26a-5p hsa-miR-26b-5p brain-miR-161 brain-miR-112 hsa-let-7d-3p hsa-miR-103a-3p hsa-miR-532-5p		Peripheral blood	–
Li et al., 2015 (18)	N = 18	N = 18	miR-644^a^ miR-450b^a^ miR-328^a^ miR-182^a^	miR-335^a^ miR-583 miR-708^a^ miR-650 miR-654^a^	miR-541 miR-663 miR-578	Blood	*GRM4*
Maffioletti et al., 2016 (27)	N = 20	N = 20	hsa-miR-199a-5p hsa-miR-24-3p hsa-miR-425-3p hsa-miR-29c-5p hsa-miR-330-3p hsa-miR-345-5p	hsa-let-7a-5p hsa-let-7d-5p has-let-7f-5p has-miR-1915-3p	hsa-miR-720 hsa-miR-140-3p hsa-miR-1973 hsa-miR-30d-5p hsa-miR-3158-3p hsa-miR-330-5p hsa-miR-378a-5p hsa-miR-1915-5p hsa-miR-1972 hsa-miR-21-3p hsa-miR-4521 hsa-miR-4793-3p hsa-miR-4440	Peripheral venous blood	*HTR2C* *MAOA* *DRD1* *CAMKK2* *NTRK3* *CLOCK* *CREB1* *GABRA2* *MTHFR* *MTHFR*
Roy et al., 2017 (28)	N = 18	N = 17	miR-124-3p			Serum from venous blood	*GRIA2* *GRIA3* *GRIA4* *NR3C1* *AKT1S1*
Sun et al., 2016 (25)	N = 32	N = 32	miR 34b-5p miR 34c-5p		miR-369–3p miR-381 miR-107	Peripheral blood leukocytes	*NOTCH1*
Wan et al., 2015 (11)	N = 32	N = 21	miR-125a-5p let-7d-3p miR-30a-5p miR-34a-5p miR-221-3p miR-29b-3p miR-10a-5p miR-375 miR-155–5p miR-33a-5p miR-139–5p	miR-451a miR-15b-5p miR-106b-5p miR-590–5p miR-185–5p		Serum	*AKT* *HTR2C* *CRHR1* *SCL1A2*

a microRNAS not indexed in software DIANA Tools, mirPath v3.0.

b (*GRIN2A*: glutamate ionotropic receptor NMDA type subunit 2A; *GRIN3A:* glutamate ionotropic receptor AMPA type subunit 3; *GRIN4A:* glutamate ionotropic receptor AMPA type subunit 4*; DISC1*: DISC1 scaffold protein; *SLC17A7:* solute carrier family 17 member 7; *GRM4*: glutamate metabotropic receptor 4; *HTR2C:* 5-hydroxytryptamine receptor 2C; *MAOA:* monoamine oxidase A; *DRD1*: dopamine receptor D1; *CAMKK2:* calcium/calmodulin-dependent protein kinase 2; *NTRK3*: neurotrophic tyrosine kinase receptor, type 3; *CLOCK:* clock homolog; *CREB1*: cAMP responsive element binding protein 1; *GABRA2*: gamma-aminobutyric acid A receptor, alpha 2; *CNR1:* cannabinoid receptor 1; *MTHFR:* 5,10-methylenetetrahydrofolate reductase NADPH; *NR3C1*: nuclear receptor subfamily 3 group C member 1; *AKT1S1*: AKT1 substrate ; *NOTCH1*: Neurogenic locus notch homolog protein 1; *AKT:* serine protein kinases; *HTR2C*: serotonin receptors; *CRHR1:* corticotrophin-releasing hormone receptor; *SCL1A2*: glutamate transporters).

The bioinformatics analysis revealed that among the up-regulated microRNAs there were 81 total pathways and 43 union pathways (data not shown), with 15 presenting a significant difference (p ≤ 0.05) (Table 4). Among the down-regulated microRNAs, 67 total and 45 union pathways were found (data not shown), with 14 presenting a significant difference (p ≤ 0.05) (Table 5).

**Table 4:** Union pathways with significant p−value of the up−regulated microRNAs in patients diagnosed with depression. The superscript numbers indicate the number of times that the microRNA is repeated among the union pathways in patients diagnosed with depression.

Union pathways	p-value	Target genes
**Transcriptional misregulation in cancer**	**<0.001**	**96**
miR-17-5p^13^	0.003	42
miR-223-3p^2^	0.026	6
miR-330-3p^2^	0.007	17
miR-34b-5p	0.040	10
miR-34a-5p^7^	5.883	68
miR-221-3p^2^	0.023	21
**Focal adhesion**	**<0.001**	**112**
miR-17-5p	<0.001	56
miR-34c-5p^2^	0.026	16
miR-34a-5p	0.046	67
miR-29b-3p^4^	<0.001	40
**Protein processing in endoplasmic reticulum**	**<0.001**	**85**
miR-345-5p	0.031	10
miR-125a-5p^2^	0.038	20
miR-30a-5p^2^	<0.001	37
miR-221-3p	0.020	21
miR-375	<0.001	25
miR-33a-5p^2^	0.028	16
**TGF-beta signaling pathway**	**0.001**	**41**
miR-17-5p	2.705	24
miR-199a-5p^2^	0.048	6
miR-24-3p	0.023	13
miR-10a-5p	0.037	10
miR-155-5p^3^	<0.001	11
**PI3K-Akt signaling pathway**	**0.002**	**98**
miR-17-5p	0.013	69
miR-29b-3p	<0.001	53
**Renal cell carcinoma**	**0.007**	**42**
miR-17-5p	2.268	26
miR-330-3p	0.019	9
miR-34a-5p	0.004	28
**Neurotrophin signaling pathway**	**0.009**	**45**
miR-17-5p	<0.001	37
miR-139-5p^2^	0.002	13
**Thyroid cancer**	**0.011**	**18**
miR-451a^4^	0.010	2
miR-17-5p	0.011	9
miR-151a-3p	0.024	4
miR-34a-5p	4.012	17
**ErbB signaling pathway**	**0.016**	**24**
miR-199a-5p	0.020	6
miR-125a-5p	<0.001	15
miR-139-5p	0.033	11
**Prolactin signaling pathway**	**0.030**	**30**
miR-17-5p	1.331	26
miR-29b-3p	0.004	15
**Signaling pathways regulating pluripotency of stem cells**	**0.034**	**52**
miR-17-5p	0.002	34
miR-155-5p	0.015	24
miR-33a-5p	0.046	14
**HTLV-I infection**	**0.034**	**138**
miR-17-5p	0.044	60
miR-24-3p	0.005	49
miR-34a-5p	0.009	85
miR-29b-3p	0.025	36
**Apoptosis**	**0.036**	**36**
miR-451a	0.034	3
miR-34c-5p	0.012	5
miR-30a-5p	0.019	17
miR-155-5p	0.002	19
**Acute myeloid leukemia**	**0.044**	**38**
miR-451a	0.032	4
miR-17-5p	0.017	17
miR-124-3p	0.011	17
miR-34a-5p	0.010	24
**mTOR signaling pathway**	**0.050**	**38**
miR-451a	0.002	6
miR-17-5p	<0.001	23
miR-223-3p	0.026	4
miR-34a-5p	0.032	24

**Table 5:** Union pathways with significant p−value of the down−regulated microRNAs in patients diagnosed with depression. The superscript numbers indicate the number of times that the microRNA is repeated among the union pathways in patients diagnosed with depression.

Union pathways	p-value	Target genes
**Non-small cell lung cancer**	**<0.001**	**32**
miR-107^4^	0.008	17
miR-103a-3p^2^	0.020	16
miR-532-5p^3^	0.027	4
let-7a-5p^10^	0.014	18
miR-451a^2^	0.010	3
miR-106b-5p^8^	0.015	12
miR-590-5p	0.014	4
**Huntington's disease**	**<0.001**	**81**
miR-26a-5p^3^	0.034	22
miR-26b-5p	0.046	38
miR-532-5p	2.118	8
let-7a-5p	0.009	39
let-7f-5p^6^	0.018	28
miR-106b-5p	0.037	21
**Renal cell carcinoma**	**<0.001**	**40**
miR-107	0.014	19
let-7a-5p	0.024	18
miR-106b-5p	0.001	17
**Signaling pathways regulating pluripotency of stem cells**	**<0.001**	**67**
miR-320a^2^	0.013	23
miR-26a-5p	0.001	29
let-7a-5p	0.024	35
miR-106b-5p	0.004	25
**Thyroid cancer**	**0.001**	**12**
let-7a-5p	0.003	10
let-7d-5p^3^	0.050	6
let-7f-5p	0.003	9
miR-451a	0.010	2
miR-106b-5p	0.005	8
**Bacterial invasion of epithelial cells**	**0.007**	**34**
miR-103a-3p	0.047	20
let-7a-5p	0.001	23
let-7f-5p	0.016	15
**Ubiquitin mediated proteolysis**	**0.008**	**71**
let-7a-5p	0.003	41
let-7d-5p	0.039	26
miR-15b-5p^3^	0.014	31
miR-106b-5p	0.005	29
**Epstein-Barr virus infection**	**0.010**	**71**
let-7f-5p	0.012	44
miR-15b-5p	0.001	50
**Glycosaminoglycan biosynthesis - chondroitin sulfate / dermatan sulfate**	**0.016**	**11**
miR-1285-5p	0.002	1
miR-26b-5p	5.410	9
miR-15b-5p	0.028	5
**Central carbon metabolism in cancer**	**0.021**	**25**
miR-320a	0.045	12
miR-107	0.026	17
miR-650	<0.001	3
**Steroid biosynthesis**	**0.023**	**7**
miR-107	<0.001	6
miR-26a-5p	0.025	3
miR-532-5p	<0.001	1
let-7d-5p	0.016	2
**MAPK signaling pathway**	**0.023**	**84**
let-7a-5p	0.009	56
let-7f-5p	0.002	47
miR-106b-5p	0.012	42
**HTLV-I infection**	**0.032**	**81**
let-7a-5p	0.026	60
miR-106b-5p	0.025	42
**PI3K-Akt signaling pathway**	**0.045**	**78**
let-7a-5p	0.035	75
let-7f-5p	0.033	63

Regarding quality assessment of the 7 studies, unfortunately, none presented high quality according to the NOS (Supplementary data). However, through the descriptive qualitative analysis, only one study had poor quality, in which the authors did not adequately describe seven of the nine microRNAs studied and thus these microRNAs were not indexed in the DIANA Tools software (Li et al., 2015).

## 4 Discussion

Based on the results of this systematic review, the microRNAs in peripheral blood that have been related to depression were compiled. Up to now, more than 70 microRNAs have been described, of which 54 had altered levels when compared to controls: 30 up-regulated and 24 down-regulated (Table 3). Based on these data, a panel of altered microRNAs in depression was created, which could contribute to the understanding of the etiology and progression of the disease (Fig. 2).

Since the discovery of microRNAs and their role as regulatory molecules, a variety of biological phenomena have been better comprehended (Dwivedi, 2011). Evidence has suggested that microRNAs may influence the etiology and pathophysiology of psychiatric disorders, such as depression (Dwivedi, 2011; Wan et al., 2015; Camkurt et al., 2015; Li et al., 2015). According to the literature, the peripheral levels of microRNAs in subjects with depression are quite susceptible to dysregulation. For example, micoRNA-320a (Camkurt et al., 2015) and microRNA-335 (Li et al., 2015) are significantly down-regulated, while microRNA-451a (Camkurt et al., 2015) and microRNA-124-3p are significantly up-regulated (Roy et al., 2017). This interaction is believed to be so relevant that the manipulation of microRNA expression has been considered a method for treating depression (Dwivedi et al., 2005). In addition, microRNA-34a-5p has been shown to be up-regulated in depressed patients (Dwivedi et al., 2005) and appears to be susceptible to the action of 7-chlorokynurenic acid (7-CTKA), a potential and rapid antidepressant (Chen et al., 1993).

It is well known that changes in the expression levels of these regulatory molecules impact gene expression, influencing various cell signaling pathways (Dwivedi, 2011). After a careful literature review, we found very little information regarding microRNAs and depression, in agreement to the study of Yuan and collaborators (Yuan et al., 2017). Therefore, microRNAs that were up and down-regulated in subjects with depression were analyzed by bioinformatics, and 29 statistically significant pathways were identified. This adds new information to the microRNAs previously studied (Fig. 2) by presenting the pathways that can be significantly influenced by the union of dysregulated microRNAs in depression. Therefore, future researches can be directed by our confirmation of relevant pathways in depression and identification of new ones that should be explored to better comprehend this pathology.

The results of the bioinformatics analysis identified genes, such as serine/threonine kinase (*AKT*), proto-oncogene B-Raf (*BRAF*), and phosphatidylinositol-4,5-bisphosphate 3-kinase catalytic subunit alpha (*PIK3CA*) (data not shown), that were targeted by several microRNAs with dysregulated expression in depressed subjects (Table 3). This interaction between target genes and microRNAs culminated in the identification of pathways that have been already related to depression, as neurotrophin signaling pathway, mammalian target of rapamycin (mTOR) signaling pathway (Table 4), phosphoinositide 3-kinase (PI3K)/Akt signaling pathway, RAS/RAF/MAPK/ERK signaling pathway, mitogen-activated protein kinase (MAPK) signaling pathway, and signaling pathways regulating pluripotency of stem cells (Table 4, Table 5). In the pathways determined by the up-regulated microRNAs, the miR-17-5p was the most frequent, appearing in 13 pathways (Table 4), and in the down-regulated microRNA the let-7a-5p was the most frequent (10 pathways) (Table 5). Therefore, both microRNAs were the focus of our discussion.

Evidence suggests that changes in serum neurotrophic levels are consistently associated with depression (Huang and Reichardt, 2001), corroborating the presence of the neurotrophic signaling pathway from up-regulated microRNAs in the bioinformatics analysis (p = 0.009) (Table 4). One of the most studied neurotrophins is the BDNF, which plays an important role in synaptic plasticity and maintenance, and neuronal survival (Dwivedi, 2009), supporting the hypothesis that alteration in neurotrophic signaling is a major factor underlying the pathophysiology of depression (Jiang et al., 2017).

Among the representative pathways obtained from the up-regulated microRNAs, the DIANA Tools software identified the mTOR signaling pathway (p = 0.05), which has been dysregulated and associated with synaptic protein deficits in patients with depression. Reinforcing this perspective, it is suggested that neurotrophin and mTOR signaling pathways are closely related, since studies indicate that BDNF activates mTOR pathway and increases α-amino-3-hydroxy-5-methyl-4-isoxazolepropionic acid (AMPA) receptor expression and function (Autry et al., 2011), enhancing synaptic plasticity and response to antidepressant treatments (Ignácio et al., 2016). We emphasize that AMPA is a receptor for glutamate that mediates fast synaptic transmission in the central nervous system (Honoré et al., 1982; Abelaira et al., 2014).

The mTOR pathway can also be influenced by the PI3K/Akt signaling pathway, as Akt activity can lead to the accumulation and activation of the mTOR complex (Ludka et al., 2016). The PI3K/Akt was identified as one of the significant signaling pathways (p = 0.002) of the up-regulated microRNAs in our study. This pathway has been associated with the neurobiology of depression (Kitagishi et al., 2012; Shi et al., 2012) and shown to be modulated by some pharmacological antidepressants (Ludka et al., 2016). Although our bioinformatics analysis identified the mTOR and PI3K/Akt signaling pathways, and both have been linked with depression, the literature still lacks information considering the regulation of miR-17-5p in both pathways (mTOR, p < 0.001; PI3K/Akt, p = 0.013).

It is known that the development of depression can be accompanied by activation of the immune system, and that antidepressant treatments can have an anti-inflammatory effect (Lee and Kim, 2006). This corroborates the presence of a pro-inflammatory cytokine, such as transforming growth factor beta (TGF-β) signaling pathway, in up-regulated microRNAs related to depression (p = 0.001) (Table 4). Indeed, recent evidence has suggested that deficiency in TGF-β signaling can occur in major depression (Caraci et al., 2018). The miR-17-5p, which we found to be up-regulated and one of the most frequent microRNA, has been shown to negatively regulate the type 2 receptor serine/threonine kinases (TGFBR2), the main receptor for TGF-β. Therefore, one can hypothesize that the up-regulation of miR-17-5p could decrease TGFBR2 activity by down-regulating TGF-β pathway and favoring a depression-like phenotype. In addition, TGF-β can activate other signaling pathways such as the PI3K/Akt and the mTOR signaling pathways (Qu et al., 2016). Although this microRNA was not statistically significant (p = 2.705) in the TGF-β signaling pathway by the bioinformatics analysis, it was up-regulated in the systematic review data. Therefore, more studies are needed to investigate the relationship among miR-17-5p, TGF-β signaling pathway, and depression.

Among the up-regulated microRNAs in subjects with depression, the focal adhesion pathway (p < 0.001) should be highlighted. This pathway presented 112 associated genes, such as the integrin subunit beta 3 (*ITGB3*) (a target of microRNA-17-5p) (Table 4). This gene is responsible for encoding the beta-3 integrin-focal adhesion protein, crucial for the activity of serotonin transporters. Thus, it has been suggested that *ITGB3* may play a key role in depression and remission of the disease (Oved et al., 2013).

Currently, it is established that microRNAs play an important regulatory role in protein processing in the endoplasmic reticulum (ER) pathway (Byrd and Brewer, 2013; Maurel and Chevet, 2013), mediating cellular unfolded protein response (UPR) (Byrd and Brewer, 2013; Bartoszewska et al., 2013) – the protein responsible for removing accumulated unbundled proteins to maintain ER functionality. Recently, a study suggested that modifications in UPR system may be associated with the development of depression (Timberlake and Dwivedi, 2015). Indeed, our results showed a significant relationship between protein processing in the ER pathway and the up-regulated microRNAs in depression (p < 0.001). It is known that a wide variety of environmental insults associated with the accumulation of misfolded proteins can compromise the structure, function, and integrity of the ER, leading to stress (Rao and Bredesen, 2004). ER stress promotes the formation of reactive oxygen species and the establishment of an oxidative stress environment, which is strongly related to neuronal changes (Michel et al., 2007), as oxidative stress causes increased lipid-peroxidation as well as DNA damage and is responsible for structural and functional neuronal and glial changes in brain regions known to play a role in the development of depression (Michel et al., 2012).

Several pathways found in our bioinformatics analysis appeared to be strongly associated with depression (Table 4, Table 5), since several mechanisms of action intersect the disease’s pathophysiology and the treatment (Brigitta, 2002). Chronic stressors (Zannas et al., 2013; Conrad, 2008) and genetic vulnerability predispose changes in the hippocampus (Rao et al., 2010), inducing the reduction of neuronal plasticity and neurogenesis, fostering the emergence of depression (Banasr and Duman, 2007). Thus, it can be hypothesized that the signaling pathways regulating pluripotency of stem cells could be indicative of neuronal reestablishment, since recent findings support that cerebral cell volume in patients with depression is lower when compared to healthy subjects (Campbell et al., 2004), perhaps due to the apoptosis pathway. This is based on the fact that antidepressant drugs that increase the availability of serotonin, such as selective serotonin reuptake inhibitors, act directly or indirectly on neurotrophins such as BDNF, stimulating neuroplasticity and neurogenesis (Taylor et al., 2005).

The MAPK signaling pathway appeared in the down-regulated microRNAs analysis as a significant pathway (p = 0.023). A positive relationship has been shown between the RAS/RAF/MAPK/ERK signaling pathway and depression (Wang et al., 2013; Mahajan et al., 2018). Studies have demonstrated that blocking the ERK signaling pathway can produce a depressive-like phenotype and block the action of antidepressants (Duman et al., 2007), and that antidepressants such as fluoxetine can increase the activity of ERK signaling pathway, alleviating depression symptoms (Qi et al., 2008). The RAS/RAF/MAPK/ERK signaling pathway is important in many brain functions, including synaptic and structural plasticity, and is crucial for neurotrophin/growth factor-mediated neuronal response (Dwivedi et al., 2009). This pathway involves a cascade of enzyme action. First, RAS recruits RAF to the cell membrane where it is phosphorylated and activated. This leads to the activation of MEK, which phosphorylates and activates ERK. After activation, extracellular-signal-regulated kinase (ERK) can phosphorylate regulatory targets in the cytosol or translocate to the nucleus (Mahajan et al., 2018; Dwivedi et al., 2009) The let-7a-5p, which was down-regulated in our systematic review and the most frequent microRNA in the down-regulated pathways (p = 0.009), has shown to inhibit the phosphorylation of ERK throughout the inhabitation of RAS (Xu et al., 2015). Let-7a-5p can modulate RAS expression, since it is complementary to multiple sequences in the 3′-untraslated region (3′UTR) of human RAS genes, and represses its expression (Johnson et al., 2005). Therefore, considering that let-7a-5p reduces the expression of RAS consequently reducing RAS/RAF/MAPK/ERK signaling pathway, which has been linked to a depression-like phenotype, the low expression of let-7a-5p shown in our systematic review seems plausible.

To better understand the biological mechanisms and to improve accuracy in the diagnosis of depression, several studies have been dedicated to investigating the microRNAs that would be associated with this pathology. Yuan and collaborators, through a systematic review, investigated circulating microRNAs as physiological indicators of the disease progression among patients diagnosed with depression. The results obtained by Yuan’s study suggested a greater number of microRNAs associated with depression (Yuan et al., 2018), when compared with our data. This discrepancy could be explained by the different search strategies and the type of studies included. As an example, we selected only case control studies since they are more accurate when considering the comparison in microRNAs expression in diseases. We had also a concern about the quality of the studies, assessed by the NOS. In addition, our study performed a bioinformatics analysis for the first time, revealing signaling pathways of the up- and down-regulated microRNAs in depression.

Some methodological details and limitations of this review should be highlighted. Initially, it should be noted that different tools for diagnosis and severity of depression were used. The heterogeneity of the chosen instruments made comparisons between studies difficult. For example, Camkurt and collaborators used the Structured Clinical Interview (SCID) for DSM-IV Disorders, Hamilton Depression Rating Scale (HDRS), and Montgomery Asberg Depression Rating Scale (MADRS) (Camkurt et al., 2015). On the other hand, Sun and collaborators used the Chinese version of DSM-IV for diagnosis and the 17-item Hamilton depression rating scale (HAMD-17) (Sun et al., 2016) (Table 2). In addition, one study did not report the method for the diagnosis of depression (Leidinger et al., 2013). However, all the other studies used widely accepted tools for diagnostic and severity measurement, whereas no study used self-reported data. Another limitation of our study is the poor quality of the articles included, as verified by the NOS. Among the three domains of the scale, the comparability domain was the one that most contributed to the low score in the star system. According to the NOS, the cases and controls should be matched and/or the confounding factors should be adjusted for in the analysis. However, none of the studies contemplated this aspect (Supplementary data).

In addition, the large diversity of identified microRNAs is a limitation of the included studies. Currently, a wide variety of microRNAs are presented in the literature but there is little reproduction of studies investigating the same molecules from different perspectives. It is necessary to investigate the behavior of regulatory molecules in populations of diverse geographic locations and submitted to different exposures to consistently delineate the profile of microRNAs. The vast majority of studies are limited to investigating the impact of microRNAs on anti-depressant treatments, rather than assessing the related mechanisms of action, which was our main interest. In addition, the data presented by the included studies show a great heterogeneity of microRNAs evaluated, using different techniques for the analysis (RT-PCR and microarray), and in many cases not presenting values for the microRNA expression, indicating only an increase or reduction of the expression levels, therefore infeasible the use of meta-analysis. Therefore, the discussion and the understanding of the baseline aspects of the pathophysiological mechanism of depression was limited. In addition, it is worth noting that although the pathways found are derived from microRNAs related to depression, the software DIANA Tools does not take into account the disease and may present pathways that are not closely related to depression.

Ultimately, it is evident that more case control studies evaluating the expression of microRNAs in depression are needed. These future clinical studies should be conducted with a careful standardization of the case control study design and data presentation of the microRNA expression. The microRNAs that have been evaluated in depression until now, and gathered by this systematic review, should be analyzed in populations from different geographic locations so that the data could be associated, establishing a more reliable set of microRNAs for depression. Additionally, it is important to enlarge the panorama of deregulated microRNAs in depression, and therefore, other microRNAs should be evaluated. Finally, the literature has agree that there is a relationship between circulating microRNAs and depression, and therefore it is important to take a step forward in understanding the target of this microRNAs, using *in vitro*, *in vivo* and *in silico* models.

In conclusion the results of our systematic review demonstrated that microRNAs are altered in depression patients; we found 54 altered microRNAs in comparison to controls. The bioinformatics analysis revealed that among the up- and down-regulated microRNAs, 29 union pathways were statistically significant. The study confirmed pathways that have been related to depression, such as neurotrophin, mTOR, PI3K/Akt, RAS/RAF/MAPK/ERK, and stem cells pluripotency regulating pathways, and presented others that have not been often related to depression, such as the endoplasmic reticulum protein-processing pathway and the focal adhesion pathway. The miR-17-5p and let-7a-5p were the most frequently found microRNAs in the pathways determined by bioinformatics. Although our study has limitations, the presented panel of microRNAs with their related pathways is a step towards understanding the complex network of microRNAs in depression.

## Funding

This study was supported by CNPq (Conselho Nacional de Desenvolvimento Científico e Tecnológico)/Brazil, Bill & Melinda Gates Foundation/USA and Ministry of Health/INCT (National Institute of Science and Technology)/Brazil (Process 401726/2015-0 APP/Call 47/2014).

## Declarations of interest

None.

## References

[bib0005] Abelaira H.M., Réus G.Z., Neotti M.V., Quevedo J. (2014). The role of mTOR in depression and antidepressant responses. Life Sci..

[bib0010] Autry A.E., Adachi M., Nosyreva E., Na E.S., Los M.F., Cheng P.F., Monteggia L.M. (2011). NMDA receptor blockade at rest triggers rapid behavioural antidepressant responses. Nature.

[bib0015] Banasr M., Duman R.S. (2007). Regulation of neurogenesis and gliogenesis by stress and antidepressant treatment. CNS Neurol. Disord. Drug Targets.

[bib0020] Bartoszewska S., Kochan K., Madanecki P., Piotrowski A., Ochocka R., Collawn J.F., Bartoszewski R. (2013). Regulation of the unfolded protein response by microRNAs. Cell. Mol. Biol. Lett..

[bib0025] Baudry A., Mouillet-Richard S., Schneider B., Launay J.M., Kellermann O. (2010). miR-16 targets the serotonin transporter: a new facet for adaptive responses to antidepressants. Science.

[bib0030] Baxter A.J., Patton G., Scott K.M., Degenhardt L., Whiteford H.A. (2013). Global epidemiology of mental disorders: what are we missing?. PLoS One.

[bib0035] Boeker M., Vach W., Motschall E. (2013). Google Scholar as replacement for systematic literature searches: good relative recall and precision are not enough. BMC Med. Res. Methodol..

[bib0040] Brigitta B. (2002). Pathophysiology of depression and mechanisms of treatment. Dialogues Clin. Neurosci..

[bib0045] Byrd A.E., Brewer J.W. (2013). Micro(RNA)managing endoplasmic reticulum stress. IUBMB Life.

[bib0050] Camkurt M.A., Acar Ş., Coşkun S., Güneş M., Güneş S., Yılmaz M.F., Tamer L. (2015). Comparison of plasma MicroRNA levels in drug naive, first episode depressed patients and healthy controls. J. Psychiatr. Res..

[bib0055] Campbell S., Marriott M., Nahmias C., MacQueen G.M. (2004). Lower hippocampal volume in patients suffering from depression: a meta-analysis. Am. J. Psychiatry.

[bib0060] Caraci F., Spampinato S.F., Morgese M.G., Tascedda F., Salluzzo M.G., Giambirtone M.C., Copani A. (2018). Neurobiological links between depression and AD: The role of TGF-β1 signaling as a new pharmacological target. Pharmacol. Res..

[bib0065] Chen J., Graham S., Moroni F., Simon R. (1993). A study of the dose dependency of a glycine receptor antagonist in focal ischemia. J. Pharmacol. Exp. Ther..

[bib0070] Conrad C.D. (2008). Chronic stress-induced hippocampal vulnerability: the glucocorticoid vulnerability hypothesis. Rev. Neurosci..

[bib0075] Duman C.H., Schlesinger L., Kodama M., Russell D.S., Duman R.S. (2007). A role for MAP kinase signaling in behavioral models of depression and antidepressant treatment. Biol. Psychiatry.

[bib0080] Dwivedi Y. (2009). Brain-derived neurotrophic factor: role in depression and suicide. Neuropsychiatr. Dis. Treat..

[bib0085] Dwivedi Y. (2011). Evidence demonstrating role of microRNAs in the etiopathology of major depression. J. Chem. Neuroanat..

[bib0090] Dwivedi Y. (2014). Emerging role of microRNAs in major depressive disorder: diagnosis and therapeutic implications. Dialogues Clin. Neurosci..

[bib0095] Dwivedi Y., Mondal A.C., Rizavi H.S., Conley R.R. (2005). Suicide brain is associated with decreased expression of neurotrophins. Biol. Psychiatry.

[bib0100] Dwivedi Y., Rizavi H.S., Zhang H., Roberts R.C., Conley R.R., Pandey G.N. (2009). Aberrant extracellular signal-regulated kinase (ERK)1/2 signalling in suicide brain: role of ERK kinase 1 (MEK1). Int. J. Neuropsychopharmacol..

[bib0105] Esquela-Kerscher A., Slack F.J. (2006). Oncomirs - microRNAs with a role in cancer. Nat. Rev. Cancer.

[bib0110] Fan H.M., Sun X.Y., Guo W., Zhong A.F., Niu W., Zhao L., Lu J. (2014). Differential expression of microRNA in peripheral blood mononuclear cells as specific biomarker for major depressive disorder patients. J. Psychiatr. Res..

[bib0115] He L., Hannon G.J. (2004). MicroRNAs: small RNAs with a big role in gene regulation. Nat. Rev. Genet..

[bib0120] Honoré T., Lauridsen J., Krogsgaard-Larsen P. (1982). The binding of [3H]AMPA, a structural analogue of glutamic acid, to rat brain membranes. J. Neurochem..

[bib0125] Huang E.J., Reichardt L.F. (2001). Neurotrophins: roles in neuronal development and function. Annu. Rev. Neurosci..

[bib0130] Ignácio Z.M., Réus G.Z., Arent C.O., Abelaira H.M., Pitcher M.R., Quevedo J. (2016). New perspectives on the involvement of mTOR in depression as well as in the action of antidepressant drugs. Br. J. Clin. Pharmacol..

[bib0135] Jiang H., Chen S., Li C., Lu N., Yue Y., Yin Y., Yuan Y. (2017). The serum protein levels of the tPA-BDNF pathway are implicated in depression and antidepressant treatment. Transl. Psychiatry.

[bib0140] Johnson S.M., Grosshans H., Shingara J., Byrom M., Jarvis R., Cheng A. (2005). RAS is regulated by the let-7 microRNA family. Cell.

[bib0145] Kitagishi Y., Kobayashi M., Kikuta K., Matsuda S. (2012). Roles of PI3K/AKT/GSK3/mTOR pathway in cell signaling of mental illnesses. Depress. Res. Treat..

[bib0150] Lee K.M., Kim Y.K. (2006). The role of IL-12 and TGF-beta1 in the pathophysiology of major depressive disorder. Int. Immunopharmacol..

[bib0155] Leidinger P., Backes C., Deutscher S., Schmitt K., Mueller S.C., Frese K. (2013). A blood based 12-miRNA signature of Alzheimer disease patients. Genome Biol..

[bib0160] Li J., Meng H., Cao W., Qiu T. (2015). MiR-335 is involved in major depression disorder and antidepressant treatment through targeting GRM4. Neurosci. Lett..

[bib0165] Li Y.J., Xu M., Gao Z.H., Wang Y.Q., Yue Z., Zhang Y.X., Wang P.Y. (2013). Alterations of serum levels of BDNF-related miRNAs in patients with depression. PLoS One.

[bib0170] Lipschitz D.L., Kuhn R., Kinney A.Y., Donaldson G.W., Nakamura Y. (2013). Reduction in salivary alpha-amylase levels following a mind-body intervention in cancer survivors--an exploratory study. Psychoneuroendocrinology.

[bib0175] Ludka F.K., Constantino L.C., Dal-Cim T., Binder L.B., Zomkowski A., Rodrigues A.L., Tasca C.I. (2016). Involvement of PI3K/Akt/GSK-3β and mTOR in the antidepressant-like effect of atorvastatin in mice. J. Psychiatr. Res..

[bib0180] Maffioletti E., Cattaneo A., Rosso G., Maina G., Maj C., Gennarelli M. (2016). Peripheral whole blood microRNA alterations in major depression and bipolar disorder. J. Affect. Disord..

[bib0185] Mahajan G.J., Vallender E.J., Garrett M.R., Challagundla L., Overholser J.C., Jurjus G., Stockmeier C.A. (2018). Altered neuro-inflammatory gene expression in hippocampus in major depressive disorder. Prog. Neuropsychopharmacol. Biol. Psychiatry.

[bib0190] Maluf D.G., Dumur C.I., Suh J.L., Scian M.J., King A.L., Cathro H. (2014). The urine microRNA profile may help monitor post-transplant renal graft function. Kidney Int..

[bib0195] Mari-Alexandre J., Sanchez-Izquierdo D., Gilabert-Estelles J., Barcelo-Molina M., Braza-Boils A., Sandoval J. (2016). miRNAs regulation and its role as biomarkers in endometriosis. Int. J. Mol. Sci..

[bib0200] Maurel M., Chevet E. (2013). Endoplasmic reticulum stress signaling: the microRNA connection. Am. J. Physiol. Cell Physiol..

[bib0205] Michel T.M., Frangou S., Thiemeyer D., Camara S., Jecel J., Nara K., Riederer P. (2007). Evidence for oxidative stress in the frontal cortex in patients with recurrent depressive disorder--a postmortem study. Psychiatry Res..

[bib0210] Michel T.M., Pülschen D., Thome J. (2012). The role of oxidative stress in depressive disorders. Curr. Pharm. Des..

[bib0215] Moher D., Liberati A., Tetzlaff J., Altman D.G., Group P. (2009). Preferred reporting items for systematic reviews and meta-analyses: the PRISMA statement. J. Clin. Epidemiol..

[bib0220] Mouillet-Richard S., Baudry A., Launay J.M., Kellermann O. (2012). MicroRNAs and depression. Neurobiol. Dis..

[bib0225] Oved K., Morag A., Pasmanik-Chor M., Rehavi M., Shomron N., Gurwitz D. (2013). Genome-wide expression profiling of human lymphoblastoid cell lines implicates integrin beta-3 in the mode of action of antidepressants. Transl. Psychiatry.

[bib0230] Qi X., Lin W., Li J., Li H., Wang W., Wang D., Sun M. (2008). Fluoxetine increases the activity of the ERK-CREB signal system and alleviates the depressive-like behavior in rats exposed to chronic forced swim stress. Neurobiol. Dis..

[bib0235] Qu Y., Zhang H., Duan J., Liu R., Deng T., Bai M., Ba Y. (2016). MiR-17-5p regulates cell proliferation and migration by targeting transforming growth factor-β receptor 2 in gastric cancer. Oncotarget.

[bib0240] Rao R.V., Bredesen D.E. (2004). Misfolded proteins, endoplasmic reticulum stress and neurodegeneration. Curr. Opin. Cell Biol..

[bib0245] Rao U., Chen L.A., Bidesi A.S., Shad M.U., Thomas M.A., Hammen C.L. (2010). Hippocampal changes associated with early-life adversity and vulnerability to depression. Biol. Psychiatry.

[bib0250] Rivera-Barahona A., Pérez B., Richard E., Desviat L.R. (2017). Role of miRNAs in human disease and inborn errors of metabolism. J. Inherit. Metab. Dis..

[bib0255] Roy B., Dunbar M., Shelton R.C., Dwivedi Y. (2017). Identification of MicroRNA-124-3p as a putative epigenetic signature of major depressive disorder. Neuropsychopharmacology.

[bib0260] Sebastiani G., Nigi L., Grieco G.E., Mancarella F., Ventriglia G., Dotta F. (2017). Circulating microRNAs and diabetes mellitus: a novel tool for disease prediction, diagnosis, and staging?. J. Endocrinol. Invest..

[bib0265] Shi H.S., Zhu W.L., Liu J.F., Luo Y.X., Si J.J., Wang S.J., Lu L. (2012). PI3K/Akt signaling pathway in the basolateral amygdala mediates the rapid antidepressant-like effects of trefoil factor 3. Neuropsychopharmacology.

[bib0270] Smalheiser N.R., Lugli G., Zhang H., Rizavi H., Cook E.H., Dwivedi Y. (2014). Expression of microRNAs and other small RNAs in prefrontal cortex in schizophrenia, bipolar disorder and depressed subjects. PLoS One.

[bib0275] Smith K. (2014). Mental health: a world of depression. Nature.

[bib0280] Song M.F., Dong J.Z., Wang Y.W., He J., Ju X., Zhang L., Lv Y.Y. (2015). CSF miR-16 is decreased in major depression patients and its neutralization in rats induces depression-like behaviors via a serotonin transmitter system. J. Affect. Disord..

[bib0285] Sun N., Lei L., Wang Y., Yang C., Liu Z., Li X., Zhang K. (2016). Preliminary comparison of plasma notch-associated microRNA-34b and -34c levels in drug naive, first episode depressed patients and healthy controls. J. Affect. Disord..

[bib0290] Taylor C., Fricker A.D., Devi L.A., Gomes I. (2005). Mechanisms of action of antidepressants: from neurotransmitter systems to signaling pathways. Cell. Signal..

[bib0295] Timberlake M.A., Dwivedi Y. (2015). Altered expression of endoplasmic reticulum stress associated genes in Hippocampus of learned helpless rats: relevance to depression pathophysiology. Front. Pharmacol..

[bib0300] Valadi H., Ekstrom K., Bossios A., Sjostrand M., Lee J.J., Lotvall J.O. (2007). Exosome-mediated transfer of mRNAs and microRNAs is a novel mechanism of genetic exchange between cells. Nat. Cell Biol..

[bib0305] Vickers K.C., Palmisano B.T., Shoucri B.M., Shamburek R.D., Remaley A.T. (2011). MicroRNAs are transported in plasma and delivered to recipient cells by high-density lipoproteins. Nat. Cell Biol..

[bib0310] Vreugdenhil E., Berezikov E. (2010). Fine-tuning the brain: MicroRNAs. Front. Neuroendocrinol..

[bib0315] Wan Y., Liu Y., Wang X., Wu J., Liu K., Zhou J., Zhang C. (2015). Identification of differential microRNAs in cerebrospinal fluid and serum of patients with major depressive disorder. PLoS One.

[bib0320] Wang C.J., Zhang Z.J., Xu Z., Shi Y.Y., Pu M.J., Zheng Z., Li L.J. (2013). Kinase gene haplotypes and gene-gene interactions in the Ras-Raf-MAPK signaling pathway: association with antidepressant remission. Int. Clin. Psychopharmacol..

[bib0325] Xie Z., Chen G., Zhang X., Li D., Huang J., Yang C. (2013). Salivary microRNAs as promising biomarkers for detection of esophageal cancer. PLoS One.

[bib0330] Xu C., Sun X., Qin S., Wang H., Zheng Z., Xu S., Ren H. (2015). Let-7a regulates mammosphere formation capacity through Ras/NF-κB and Ras/MAPK/ERK pathway in breast cancer stem cells. Cell Cycle.

[bib0335] Yuan H., Mischoulon D., Fava M., Otto M.W. (2017). Circulating microRNAs as biomarkers for depression: Many candidates, few finalists. J. Affect. Disord..

[bib0340] Yuan H., Mischoulon D., Fava M., Otto M.W. (2018). Circulating microRNAs as biomarkers for depression: many candidates, few finalists. J. Affect. Disord..

[bib0345] Zannas A.S., McQuoid D.R., Payne M.E., Steffens D.C., MacFall J.R., Ashley-Koch A., Taylor W.D. (2013). Negative life stress and longitudinal hippocampal volume changes in older adults with and without depression. J. Psychiatr. Res..

[bib0350] Zernecke A., Bidzhekov K., Noels H., Shagdarsuren E., Gan L., Denecke B. (2009). Delivery of microRNA-126 by Apoptotic Bodies Induces CXCL12-dependent Vascular Protection. Science Signaling. 2:ra81.

[bib0355] Zhang Y., Huang B., Wang H.Y., Chang A., Zheng X.F.S. (2017). Emerging role of MicroRNAs in mTOR signaling. Cell. Mol. Life Sci..

