# Supplementary material for: MicroRNAs expressed in depression and their associated pathways: A systematic review and a bioinformatics analysis
Source: J Chem Neuroanat. 2019 Oct;100:101650. doi: 10.1016/j.jchemneu.2019.101650 (PMC6996133; doi:10.1016/j.jchemneu.2019.101650)
Supplement: Supplementary file 1 [file mmc1.docx]

**Table S1:** Results of the NOS to verify the quality of the included studies.

|  | **Selection** | | | | **Comparability** | | **Exposure** | | |
| --- | --- | --- | --- | --- | --- | --- | --- | --- | --- |
|  | **1** | **2** | **3** | **4** | **1.a** | **1.b** | **1** | **2** | **3** |
| Camkurt et al. 2015^(1)^ | a | a | a | a | - | - | b | a | b |
| Leidinger et al., 2013^(2)^ | c | b | b | b | - | - | e | b | b |
| Li et al., 2015^(3)^ | a | a | c | b | - | - | b | b | b |
| Maffioletti et al., 2017 ^(4)^ | a | b | c | a | - | - | b | a | b |
| Roy et al., 2017^(5)^ | a | b | c | a | - | - | b | a | b |
| Sun et al., 2016^(6)^ | a | a | a | a | - | - | b | a | b |
| Wan et al., 2015^(7)^ | a | b | c | a | - | - | b | a | b |

**References:**

1. Camkurt MA, Acar Ş, Coşkun S, Güneş M, Güneş S, Yılmaz MF, et al. (2015) Comparison of plasma MicroRNA levels in drug naive, first episode depressed patients and healthy controls. *J Psychiatr Res*. **69** , 67-71.

2. Leidinger P, Backes C, Deutscher S, Schmitt K, Mueller SC, Frese K, et al. (2013) A blood based 12-miRNA signature of Alzheimer disease patients. *Genome Biol.* **14** , R78.

3. Li J, Meng H, Cao W, Qiu T. (2015) MiR-335 is involved in major depression disorder and antidepressant treatment through targeting GRM4. *Neurosci Lett.* **606** , 167-172.

4. Maffioletti E, Cattaneo A, Rosso G, Maina G, Maj C, Gennarelli M, et al. (2016) Peripheral whole blood microRNA alterations in major depression and bipolar disorder. *J Affect Disord.* **200** , 250-258.

5. Roy B, Dunbar M, Shelton RC, Dwivedi Y. (2017) Identification of MicroRNA-124-3p as a Putative Epigenetic Signature of Major Depressive Disorder. *Neuropsychopharmacology.* **42** , 864-875.

6. Sun N, Lei L, Wang Y, Yang C, Liu Z, Li X, et al. (2016) Preliminary comparison of plasma notch-associated microRNA-34b and -34c levels in drug naive, first episode depressed patients and healthy controls. *J Affect Disord.* **194** , 109-114.

7. Wan Y, Liu Y, Wang X, Wu J, Liu K, Zhou J, et al. (2015) Identification of differential microRNAs in cerebrospinal fluid and serum of patients with major depressive disorder. *PLoS One.* **10** , e0121975.

**Newcastle - Ottawa quality assessment scale case control studies**

Note: A study can be awarded a maximum of one star for each numbered item within the Selection and Exposure categories. A maximum of two stars can be given for Comparability.

**Selection:**

1) Is the case definition adequate?
a) yes, with independent validation Ø
b) yes, eg record linkage or based on self reports
c) no description

2) Representativeness of the cases
a) consecutive or obviously representative series of cases Ø
b) potential for selection biases or not stated

3) Selection of Controls
a) community controls Ø
b) hospital controls
c) no description

4) Definition of Controls
a) no history of disease (endpoint) Ø
b) no description of source

**Comparability:**

1) Comparability of cases and controls on the basis of the design or analysis
a) study controls for _______________ (Select the most important factor.) Ø
b) study controls for any additional factor Ø (This criteria could be modified to indicate specific control for a second important factor.)

**Exposure:**

1) Ascertainment of exposure
a) secure record (eg surgical records) Ø
b) structured interview where blind to case/control status Ø
c) interview not blinded to case/control status
d) written self report or medical record only
e) no description

2) Same method of ascertainment for cases and controls
a) yes Ø
b) no

3) Non-Response rate
a) same rate for both groups Ø
b) non respondents described
c) rate different and no designation

**Coding manual for case-control studies
Selection:**

1)  Is the Case Definition Adequate?
a)  Requires some independent validation (e.g. >1 person/record/time/process to extract information, or reference to primary record source such as x-rays or medical/hospital records) Ø
b)  Record linkage (e.g. ICD codes in database) or self-report with no reference to primary record
c)  No description

2)  Representativeness of the Cases
a)  All eligible cases with outcome of interest over a defined period of time, all cases in a defined catchment area, all cases in a defined hospital or clinic, group of hospitals, health maintenance organisation, or an appropriate sample of those cases (e.g. random sample) Ø
b)  Not satisfying requirements in part (a), or not stated.

3)  Selection of Controls

This item assesses whether the control series used in the study is derived from the same population as the cases and essentially would have been cases had the outcome been present.
a)  Community controls (i.e. same community as cases and would be cases if had outcome) Ø
b)  Hospital controls, within same community as cases (i.e. not another city) but derived from a hospitalised population
c)  No description

4)  Definition of Controls
a)  If cases are first occurrence of outcome, then it must explicitly state that controls have no history of this outcome. If cases have new (not necessarily first) occurrence of outcome, then controls with previous occurrences of outcome of interest should not be excluded. Ø
b)  No mention of history of outcome

**Comparability:**

1) Comparability of Cases and Controls on the Basis of the Design or Analysis

A maximum of 2 stars can be allotted in this category
Either cases and controls must be matched in the design and/or confounders must be adjusted for in the analysis. Statements of no differences between groups or that differences were not statistically significant are not sufficient for establishing comparability. Note: If the odds ratio for the exposure of interest is adjusted for the confounders listed, then the groups will be considered to be comparable on each variable used in the adjustment.
There may be multiple ratings for this item for different categories of exposure (e.g. ever vs. never, current vs. previous or never)
Age = Ø Other controlled factors = Ø

**Exposure:**

1) Ascertainment of Exposure
Allocation of stars as per rating sheet

2) Non-Response Rate
Allocation of stars as per rating sheet
